# Putting an ultrahigh concentration of amine groups into a metal–organic framework for CO_2_ capture at low pressures†
†Electronic supplementary information (ESI) available: Experimental details, methods, additional discussions, thermogravimetry curves, PXRD patterns, and spectroscopy characterizations, as well as additional isotherms. See DOI: 10.1039/c6sc00836d



**DOI:** 10.1039/c6sc00836d

**Published:** 2016-07-13

**Authors:** Pei-Qin Liao, Xun-Wei Chen, Si-Yang Liu, Xu-Yu Li, Yan-Tong Xu, Minni Tang, Zebao Rui, Hongbing Ji, Jie-Peng Zhang, Xiao-Ming Chen

**Affiliations:** a MOE Key Laboratory of Bioinorganic and Synthetic Chemistry , School of Chemistry and Chemical Engineering , Sun Yat-Sen University , Guangzhou 510275 , China . Email: zhangjp7@mail.sysu.edu.cn

## Abstract

Tremendous efforts have been devoted to increasing the CO_2_ capture performance of porous materials, especially for low CO_2_ concentration environments. Here, we report that hydrazine can be used as a diamine short enough to functionalize the small-pore metal–organic framework [Mg_2_(dobdc)] (H_4_dobdc = 2,5-dihydroxyl-1,4-benzenedicarboxylic acid). By virtue of the ultrahigh concentration of free amine groups (6.01 mmol g^–1^ or 7.08 mmol cm^–3^) capable of reversible carbamic acid formation, the new material [Mg_2_(dobdc)(N_2_H_4_)_1.8_] achieves a series of new records for CO_2_ capture, such as single-component isotherm uptakes of 3.89 mmol g^–1^ or 4.58 mmol cm^–3^ at the atmospheric CO_2_ concentration of 0.4 mbar at 298 K and 1.04 mmol g^–1^ or 1.22 mmol cm^–3^ at 328 K, as well as more than a 4.2 mmol g^–1^ or 4.9 mmol cm^–3^ adsorption/desorption working capacity under dynamic mixed-gas conditions with CO_2_ concentrations similar to those in flue gases and ambient air.

## Abstract 2

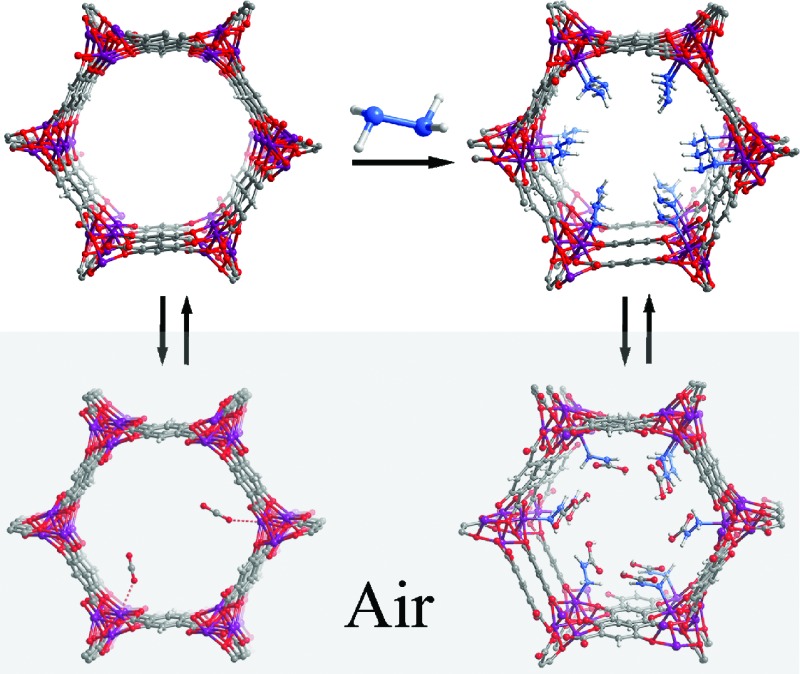
Hydrazine can be grafted in CPO-27-Mg/MOF-74-Mg to provide an ultrahigh concentration of amine groups on the pore surface, giving an exceptionally high CO_2_ capture performance, especially at extremely low pressures.

## Introduction

Applications of porous materials for capturing CO_2_ from flue gas (*ca.* 150 mbar) and from air (*ca.* 0.4 mbar) have become an increasingly attractive area of research due to their importance in many applications related to the environment, energy and health.^1–7^ Compared with post-combustion capture, direct air capture (DAC) is more challenging, since the CO_2_ adsorption ability of most porous materials is low at trace CO_2_ concentrations.^8^

Among the various types of adsorbents, porous coordination polymers (PCPs) or metal–organic frameworks (MOFs) with designable and modifiable pore surfaces are very attractive for CO_2_ capture.^9–50^ To increase the CO_2_ uptake at extremely low pressures, incorporating alkylamine groups on the pore surface is the most effective strategy so far,^46–50^ because of the chemisorption of CO_2_ associated with the formation of zwitterionic carbamate or carbamic acid. For instance, [Mg_2_(dobpdc)(eda)_1.6_] (eda-Mg_2_(dobpdc), eda = ethylenediamine, H_4_dobpdc = 4,4′-dihydroxy-(1,1′-biphenyl)-3,3′-dicarboxylic acid)^48^ holds the records for gravimetric and volumetric CO_2_ adsorption capacities with 2.85 mmol g^–1^ and 2.72 mmol cm^–3^, respectively, at 298 K and 0.4 mbar, this is because it has a high concentration of free amine groups at 3.86 mmol g^–1^ or 3.69 mmol cm^–3^. Obviously, the CO_2_ adsorption does not reach saturation at this condition, which is ascribed to the strong intermolecular hydrogen bonds between two adjacent amine groups that need to be opened by higher pressure CO_2_.^47,51^ As a consequence, its CO_2_ uptake at 0.4 mbar sharply drops to 0.120 mmol g^–1^ or 0.115 mmol cm^–3^ at a slightly higher temperature of 323 K. To overcome this problem, replacing the eda molecule with an even shorter diamine can be a good strategy, which not only reduces the intermolecular hydrogen bonding locks but also reduces the adsorbent weight.

Hydrazine (N_2_H_4_) can be considered as the smallest/shortest diamine, and its basicity is only slightly weaker than that of alkylamines (Table S1†).^52^ It should be noted that the long/large length/size of eda and its derivatives is the main reason for choosing the large-pore [Mg_2_(dobpdc)] instead of the simpler small-pore [Mg_2_(dobdc)] (H_4_dobdc = 2,5-dihydroxyl-1,4-benzenedicarboxylic acid) as a host framework.^49^ Nevertheless, to the best of our knowledge, an N_2_H_4_-grafted PCP has not been reported so far. Herein, we show that N_2_H_4_ can be used to functionalize the prototypical small-pore PCP [Mg_2_(dobdc)] without the formation of the intermolecular amine–amine interaction, achieving an ultrahigh concentration of useful amine groups in both the gravimetric and volumetric points of view, as well as a series of new benchmark CO_2_ capture performances, ranging from air to flue-gas conditions.

## Results and discussion

**Fig. 1:**
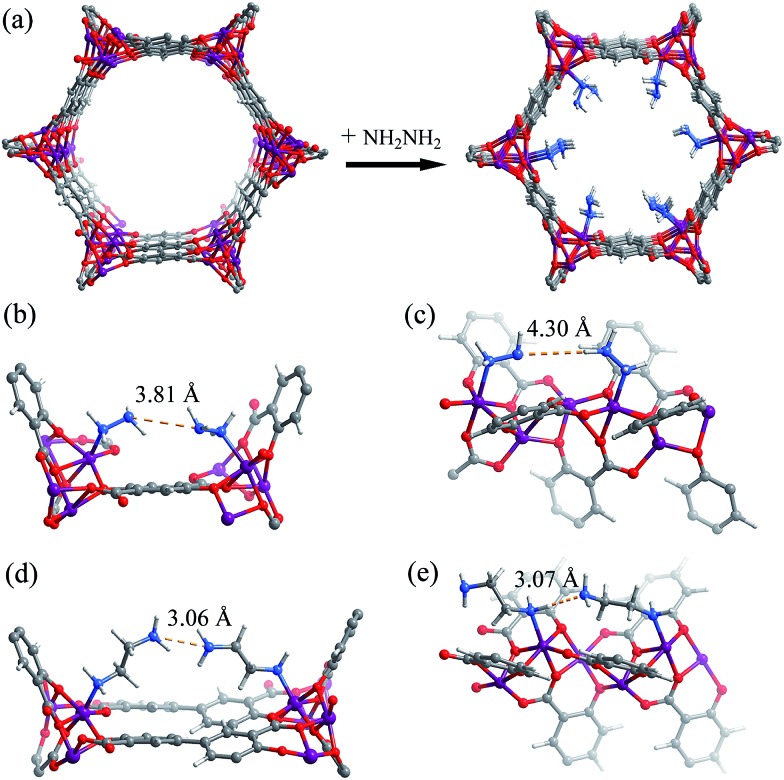
(a) Comparison of the structures of [Mg_2_(dobdc)] (drawn from the reported crystal structure) and [Mg_2_(dobdc)(N_2_H_4_)_2_] (simulated from PDFT). (b–e) Comparison of the shortest possible intermolecular hydrogen bonding in [Mg_2_(dobdc)(N_2_H_4_)_2_] and [Mg_2_(dobpdc)(eda)_2_] (obtained using a PDFT simulation, in which the flexibility of diamines was considered). (b) [Mg_2_(dobdc)(N_2_H_4_)_2_] on the *a*–*b* plane, (c) [Mg_2_(dobdc)(N_2_H_4_)_2_] along the *c*-axis, (d) [Mg_2_(dobpdc)(eda)_2_] on the *a*–*b* plane, and (e) [Mg_2_(dobpdc)(eda)_2_] along the *c*-axis.

The hypothetical structure of [Mg_2_(dobdc)(N_2_H_4_)_2_], *i.e.* [Mg_2_(dobdc)] fully functionalized by one N_2_H_4_ molecule per Mg(ii) ion, was simulated using molecular mechanics (MM) combined with periodic density functional theory (PDFT). During structural optimization, the host framework and N_2_H_4_ molecules were considered as rigid and flexible, respectively. The simulation results showed that the void ratio of [Mg_2_(dobdc)(N_2_H_4_)_2_] remains at 36.5%, and the smallest aperture diameter is *ca.* 4.8 Å (Fig. 1a), which should be enough for the diffusion and accommodation of CO_2_ molecules. Furthermore, the shortest possible intermolecular N···N separations between pairs of adjacent amine groups on the *ab*-plane and along the *c*-axis were simulated as 3.81 and 4.30 Å, respectively (Fig. 1b and c), which are much longer than the values for typical N–H···N hydrogen bonds. For comparison, we also simulated the structure of eda-Mg_2_(dobpdc), giving relatively short N···N separations of 3.06 and 3.07 Å on the *ab*-plane and along the *c*-axis, respectively (Fig. 1d and e), which can be assigned as a kind of weak hydrogen bonding.^53^

Yellow microcrystalline [Mg_2_(dobdc)] (**1**) was suspended in anhydrous toluene under N_2_ atmosphere at room temperature, and upon the addition of a toluene solution of anhydrous N_2_H_4_, the colour of the solid instantaneously changed to light yellow. The light yellow product was washed with toluene and then successively soaked in hexane and methanol to remove the excess N_2_H_4_. Elemental analysis gave a chemical formula of [Mg_2_(dobdc)(N_2_H_4_)_1.8_] (**1a**) for the light yellow product. Pawley refinements of the powder X-ray diffraction (PXRD) patterns showed similar unit-cell parameters for **1** and **1a** and confirmed the retention of framework integrity after N_2_H_4_ grafting (Fig. S1†). PXRD and thermogravimetry-mass spectrometry analyses showed that **1a** can be fully activated at 403 K (Fig. S1 and S2†), with negligible release of the N_2_H_4_ molecules. N_2_ sorption isotherms measured at 77 K gave apparent Langmuir surface areas of 1925 and 1012 m^2^ g^–1^ and pore volumes of 0.67 and 0.30 cm^3^ g^–1^, respectively, for **1** and **1a** (Fig. S3†). Considering that their crystallographic pore volumes are 0.66 and 0.31 cm^3^ g^–1^, respectively, the N_2_ sorption data further confirmed the good sample crystallinity and purity, as well as the chemical formula of **1a** derived from elemental analysis. **1a** possesses a high concentration of surface-appended N_2_H_4_ molecules of 6.01 mmol g^–1^ or 7.08 mmol cm^–3^.

**Fig. 2:**
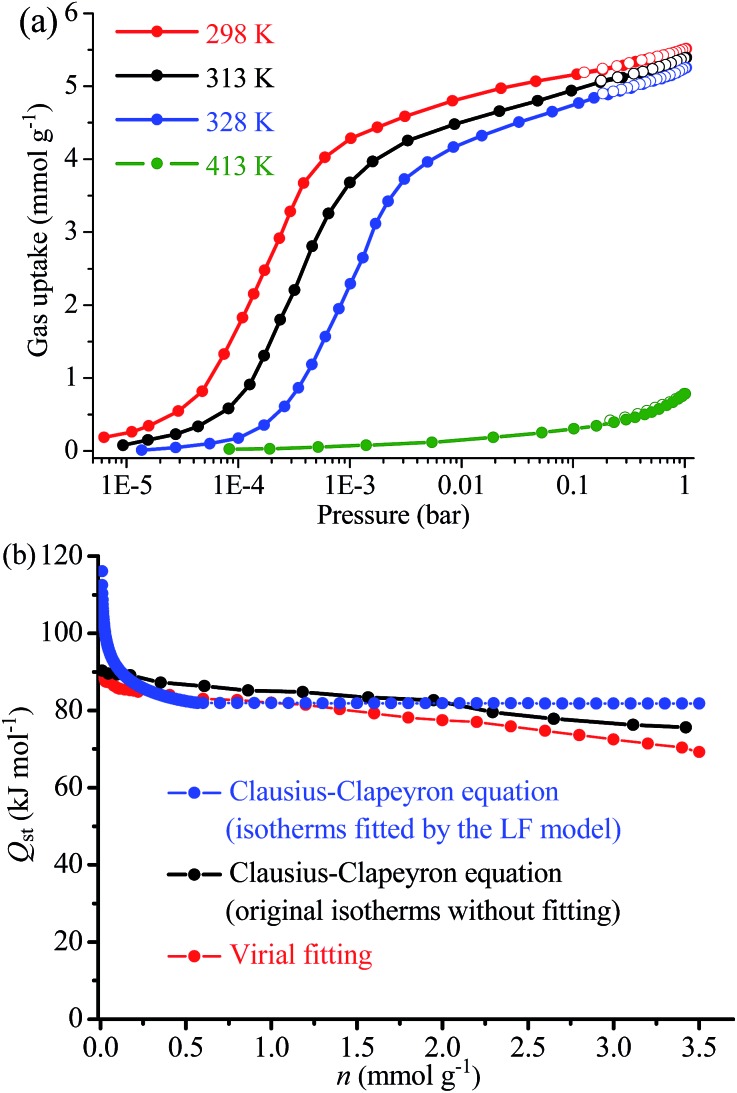
(a) CO_2_ adsorption (solid) and desorption (open) isotherms measured at 298, 313, 328 and 413 K and (b) the coverage-dependent CO_2_ adsorption enthalpy of **1a**.

At 298 K and 1 bar (Fig. 2a), **1a** showed a CO_2_ uptake of 5.51 mmol g^–1^ or 6.49 mmol cm^–3^, corresponding to 1.65 CO_2_ molecules per formula unit or 0.919 CO_2_ molecule per N_2_H_4_ molecule, which are lower than the values for **1** (8.04 mmol g^–1^ or 7.40 mmol cm^–3^) (Table S2†), this is because the relatively large pore volume of **1** can accommodate additional physically adsorbed CO_2_. At 0.15 bar, the gravimetric uptake of **1a** (5.18 mmol g^–1^) is still lower than that of **1** (5.71 mmol g^–1^). However, the volumetric uptake of **1a** (6.10 mmol cm^–3^) is 17% higher than that of **1** (5.25 mmol cm^–3^), illustrating the stronger CO_2_ binding affinity of the amine groups. At a much lower pressure of 0.4 mbar, corresponding to the CO_2_ concentration in air, **1a** showed an exceptionally high CO_2_ uptake of 3.89 mmol g^–1^ or 4.58 mmol cm^–3^, which is not only a drastic improvement over **1** (0.09 mmol g^–1^ or 0.08 mmol cm^–3^), but also obviously higher than the previous records (Table S2†). The CO_2_ sorption kinetics of **1a** were also evaluated using thermogravimetric analysis under dry air at 298 K. A CO_2_ uptake of 3.66 mmol g^–1^ or 16.1 wt% (3.71 mmol g^–1^ or 16.3 wt% for the single-component CO_2_ adsorption isotherm at 0.4 mbar and 298 K) was observed after *ca.* 950 min by switching the atmosphere from pure N_2_ at 403 K to dry air at 298 K. The equilibrium time is much longer than that at higher CO_2_ partial pressures, being similar to known examples such as eda-Mg_2_(dobpdc) (1000 min, Fig. S4†).^12,48^ Interestingly, even at a higher temperature of 328 K, **1a** still exhibits a relatively high CO_2_ uptake of 1.04 mmol g^–1^ or 1.22 mmol cm^–3^ at 0.4 mbar, which is 2–3 times higher than the previous records (Table S3†).

**Fig. 3:**
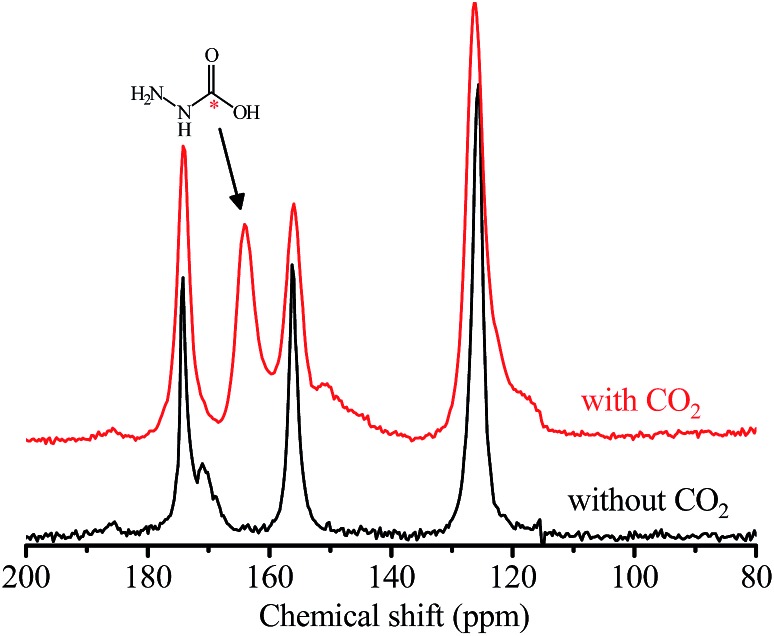
Solid-state ^13^C NMR spectra of **1a** with and without adsorbed CO_2_. An asterisk (*) marks the carbon atom of CO_2_.

The coverage-dependent CO_2_ adsorption enthalpy (*Q*_st_) of **1a** was calculated using the Clausius–Clapeyron equation and the Virial fitting method using isotherms measured at 298, 313, and 328 K (Fig. 2b, S5 and S6†). Among various isotherm models, only the Langmuir–Freundlich (LF) one gave a fair fitting. Based on the Clausius–Clapeyron equation, the Langmuir–Freundlich isotherms gave a very high *Q*_st_ of 118 kJ mol^–1^ at zero-coverage, while the original isotherms (without data fitting) gave near-zero-coverage *Q*_st_ of 90 kJ mol^–1^. These *Q*_st_ values are higher than the values of most reported materials (Table S2†) and indicative of chemisorption. On the other hand, the Virial fitting gave similar results as the Clausius–Clapeyron equation using original isotherms. *In situ* infrared (IR) spectra of **1a** showed that the primary amine peaks at 3330 and 3287 cm^–1^ weakened under CO_2_ atmosphere. The residue primary amine groups can be assigned to those anchoring on the Mg(ii) sites. Meanwhile, two new absorption peaks appeared at 3397 and 1250 cm^–1^. The former new peak may arise from the stretching band of the N–H bond and/or carboxylic O–H bond, while the latter can be assigned to the stretching band of the C–N bond of carbamic acid. The peak for the asymmetric deformation of the ammonium group at ∼2200 cm^–1^ was invisible, indicating the absence of proton transfer, which is consistent with the relatively long intermolecular separation of hydrazine molecules (Fig. S7†).^54,55^ After being heated under vacuum at 403 K, this peak disappeared, indicating the chemisorption of CO_2_ is reversible. The formation of carbamic acid was further confirmed *via* ^13^C NMR measurements with the observation of a broad resonance peak centered at *δ* = 162 ppm (Fig. 3).^22^

**Fig. 4:**
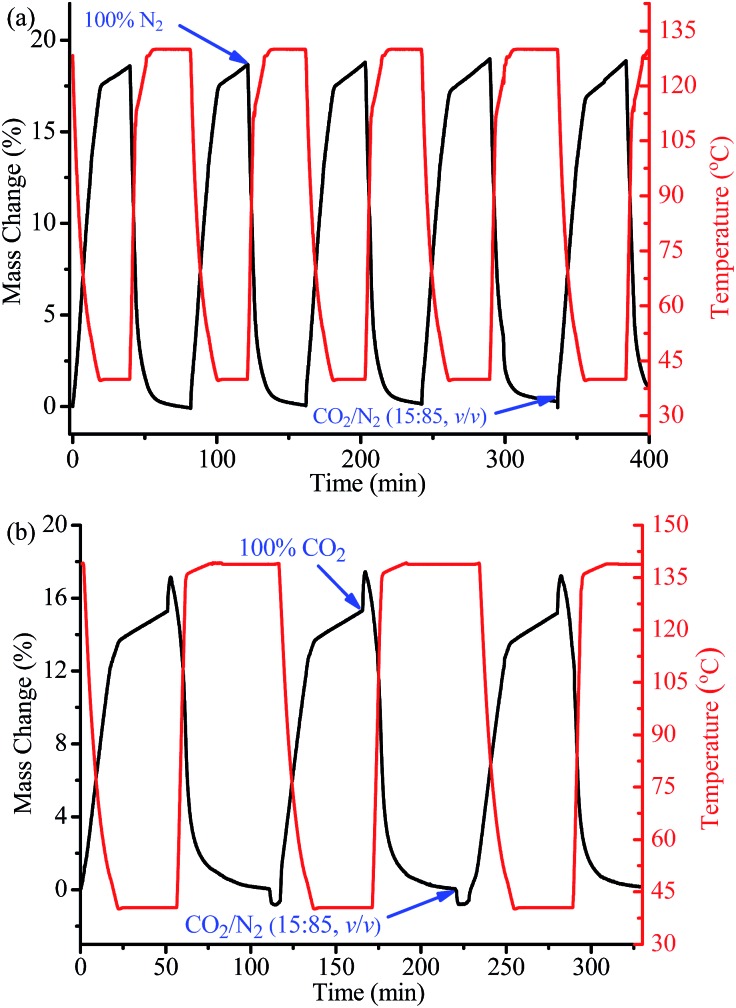
Repeated adsorption–desorption kinetics for **1a** (a) between a 15 : 85 CO_2_/N_2_ (v/v) flow at 313 K and a pure N_2_ flow at 403 K and (b) between a 15 : 85 CO_2_/N_2_ (v/v) flow at 313 K and a pure CO_2_ flow at 413 K.

The CO_2_ adsorption and desorption behaviours of **1a** under mixed-gas and kinetic conditions were analyzed *via* thermogravimetry (Fig. 4), in which the sample was blown repeatedly under conditions between a 15 : 85 CO_2_/N_2_ (v/v) mixture at 313 K (the typical flue-gas environment) and a pure N_2_ flow (a typical regeneration method for temperature-vacuum swing adsorption (TVSA) like process) at 403 K. It should be noted that the regeneration temperature is lower than for other alkylamine functionalized PCPs. By switching from pure N_2_ at 403 K to the mixed gas and allowing the temperature to automatically decrease to 313 K after 17 min, a weight increase of 17.0% was observed, corresponding to volumetric CO_2_ uptake of 3.86 mmol g^–1^ or 4.55 mmol cm^–3^, which is 78% of the single-component isotherm uptake at 313 K and 0.15 bar. The CO_2_ uptake further increased to 18.7% (4.25 mmol g^–1^ or 5.01 mmol cm^–3^, *i.e.*, 87% of the isotherm value) by maintaining the temperature at 313 K for an additional 23 min. Although the adsorption and desorption processes for **1a** were not allowed to reach equilibrium, as its relatively small pores are not beneficial for fast guest diffusion, the CO_2_ adsorption/desorption working capacities obtained here are much higher than all the reported values for other materials under similar conditions and time scales (Table S4†). After 5 such adsorption–desorption cycles, there was nearly no change in the adsorption capacity, illustrating the high thermal stability and good CO_2_ adsorption–desorption reversibility of **1a**, as indicated by the thermogravimetry-mass spectrometry analyses (Fig. S2†).

A pure temperature swing adsorption (TSA) process for **1a** was further carried out, and a working capacity of 3.52 mmol g^–1^ or 4.15 mmol cm^–3^ was obtained between the adsorption in a 15 : 85 CO_2_/N_2_ (v/v) mixture at 313 K for 48 min and the desorption in a pure CO_2_ flow at 413 K (optimized) for 54 min. It should be noted that, even though it was obtained in a relatively short time which did not allow the adsorption/desorption to reach equilibration, this working capacity is higher than previously reported values obtained in similar and longer adsorption/desorption times (Table S4†). To evaluate the regeneration energy for CO_2_ desorption, the heat capacity of **1a** was quantified *via* differential scanning calorimetry. About –200 J g^–1^ was evolved as the material was cooled from 413 to 313 K (Fig. S8†). With these data (see calculation method in the ESI†), approximately 3.02 MJ of energy would be required to regenerate 1 kg of CO_2_ from **1a** (Fig. 4b).^49,56^ The regeneration energy for CO_2_ desorption from **1a** (3.02 MJ kg^–1^) is higher than those of mmeda-Mg_2_(dobpdc) (2.34 MJ kg^–1^) and state-of-the-art amine-based solutions (2.6 MJ kg^–1^), but lower than that of monoethanolamine (3.5 MJ kg^–1^).^49^

**Fig. 5:**
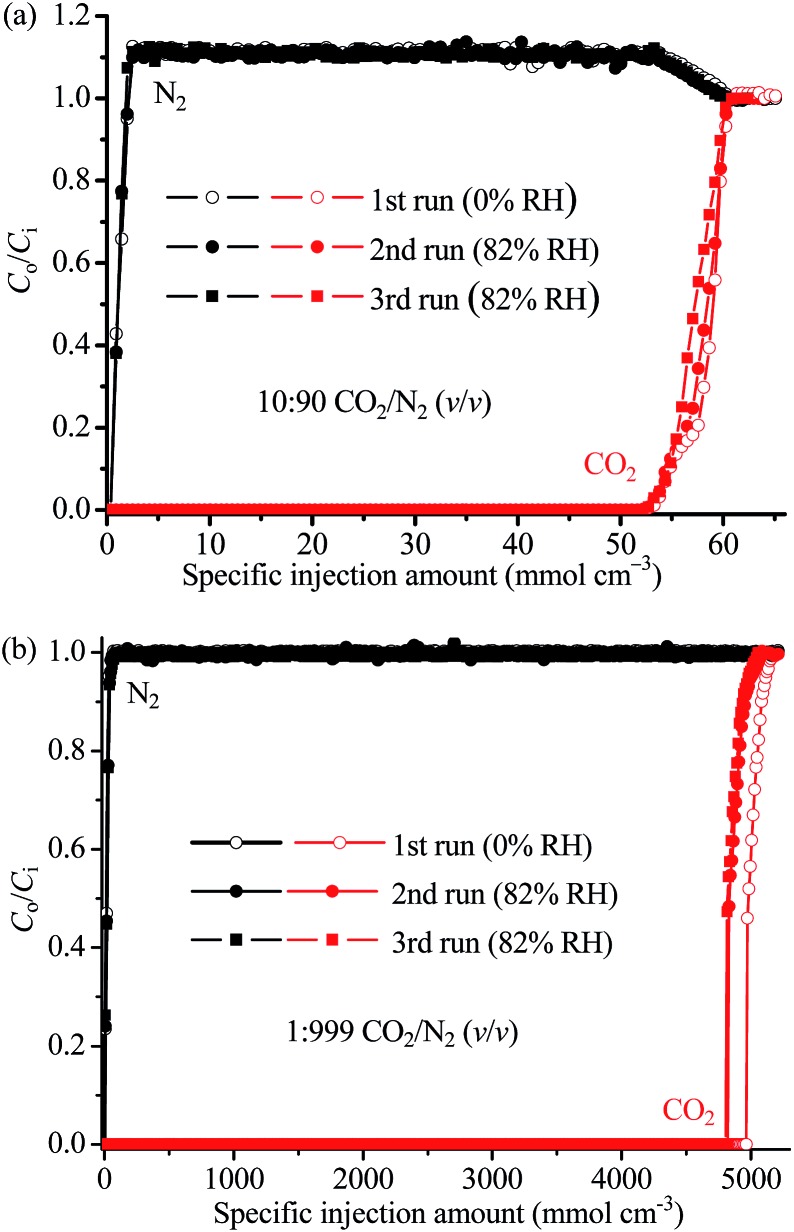
Repeated breakthrough curves of the **1a** column operated at 1 bar. (a) 10 : 90 CO_2_/N_2_ (v/v) at 313 K. (b) 1 : 999 CO_2_/N_2_ (v/v) mixture at 298 K. Lines are drawn to guide eyes. *C*_i_ and *C*_o_ are the concentrations of each gas at the inlet and outlet, respectively.

To simulate more practical CO_2_ capture applications, we also measured the column breakthrough curves of **1a** using a binary 10 : 90 CO_2_/N_2_ (v/v) mixture at 313 K and 1 bar. As shown in Fig. 5a and S9a,† the breakthrough point (outlet concentration > detection limit) of CO_2_ was observed at a mixture injection amount of 44.3 mmol g^–1^ or 52.2 mmol cm^–3^, exceeding all the reported values even measured at a lower temperature of 298 K (Table S5,† note that a lower temperature generally leads to a higher adsorption amount, at the same pressure), including 42.2 mmol g^–1^ or 38.8 mmol cm^–3^ for **1** and 25.5 mmol g^–1^ or 50.2 mmol cm^–3^ for the recently discovered microporous copper silicate SGU-29.^7^ After that, the outlet CO_2_ concentration rose gradually to the inlet value at 51.6 mmol g^–1^ or 60.8 mmol cm^–3^. The capability of **1a** for trace CO_2_ capture was investigated by column breakthrough tests using a 1 : 999 CO_2_/N_2_ (*i.e.* 1000 ppm CO_2_) mixture at 298 K (Fig. 5b). The CO_2_ breakthrough and saturation points were observed at 4210 and 4400 mmol g^–1^, or 4960 and 5180 mmol cm^–3^, respectively.

The CO_2_ uptake of the adsorbent **1a** in the columns can be calculated (see ESI† for calculation method) as 4.89 and 4.22 mmol g^–1^, or 5.76 and 4.97 mmol cm^–3^, for the 10 : 90 and 1 : 999 CO_2_/N_2_ mixtures, respectively, which are both *ca.* 98% of the values obtained from the single-component adsorption isotherms (4.97 mmol g^–1^ or 5.85 mmol cm^–3^ at 313 K and 0.1 bar, 4.30 mmol g^–1^ or 5.07 mmol cm^–3^ at 298 K and 1 mbar). Suffering from weak CO_2_/N_2_ selectivity and/or low adsorption rate, many adsorbents show a much lower CO_2_ uptake under mixed-gas conditions compared to single-component adsorption isotherms (Table S5†). The exceptionally high adsorption efficiency of **1a** implies that its CO_2_ diffusion rate and CO_2_/N_2_ selectivity are high enough under such mixed-gas breakthrough conditions.

We also tested the CO_2_ capture performances and stabilities of the columns under high humidity.^9–13^ After the breakthrough curves reached equilibrium under the above mentioned dry conditions (0% RH, RH = relative humidity), the columns were activated under He flow at 403 K, cooled down to the measurement temperature of 313 K, saturated using humid He (82% RH), and then switched to humid CO_2_/N_2_ mixtures (82% RH, no change to other parameters) to start a new breakthrough experiment. Remarkably, the breakthrough curve for the humid 10 : 90 CO_2_/N_2_ mixture almost overlapped with that measured at the dry condition (Fig. 5a). For the humid 1 : 999 CO_2_/N_2_ mixture, the CO_2_ breakthrough point slightly (2.6% compared with that observed for the dry mixture) shortened to 4100 mmol g^–1^ or 4830 mmol cm^–3^ (Fig. 5b and S9b†). These observations indicated that water has little effect on the CO_2_ adsorption capacity of **1a**, even when the CO_2_ concentration is extremely low. It should be noted that H_2_O can strongly compete with CO_2_ for the adsorption sites in most porous materials, and only a handful of examples have demonstrated such a high waterproof CO_2_ capture performance (Table S5†).

As shown in Fig. 5, the same activation/breakthrough procedures were applied to the columns once again, and the breakthrough curves almost overlapped with each other, indicating the high stability of the adsorbent **1a** and the column even under high humidity. This also indicates that **1a** is particularly selective for CO_2_ over water, and confirms that the grafted N_2_H_4_ molecules were not replaced by water molecules.

## Conclusions

In summary, we demonstrated that the classical small-pore PCP [Mg_2_(dobdc)] can be modified by the shortest diamine N_2_H_4_ to obtain a very powerful CO_2_ adsorbent [Mg_2_(dobdc)(N_2_H_4_)_1.8_]. Thanks to the ultrahigh concentration of amine groups and the chemisorption of CO_2_ associated with carbamic acid formation, this material exhibited exceptionally high CO_2_ adsorption capacity at low pressures (from 0.4 mbar to 150 mbar) and a wide range of temperatures (from 298 to 328 K), well exceeding previous records. More importantly, these CO_2_ adsorption performances can be maintained under mixed-gas kinetic conditions, even in the presence of high humidity.
