# Supplementary material for: Putting an ultrahigh concentration of amine groups into a metal–organic framework for CO2 capture at low pressures
Source: Chem Sci. 2016 Jul 13;7(10):6528–33. doi: 10.1039/c6sc00836d (PMC5125374; doi:10.1039/c6sc00836d)
Supplement: Supplementary file 1 [file SC-007-C6SC00836D-s001.pdf]

Electronic Supplementary Material (ESI) for Chemical Science.
This journal is © The Royal Society of Chemistry 2016

ESI

Putting ultrahigh-concentration amine groups into a metal–organic framework for CO_2 capture at low pressures†

Pei-Qin Liao, Xun-Wei Chen, Si-Yang Liu, Xu-Yu Li, Yan-Tong Xu, Minni Tang, Zebao Rui, Hongbing Ji, Jie-Peng Zhang* and Xiao-Ming Chen

MOE Key Laboratory of Bioinorganic and Synthetic Chemistry, School of Chemistry and Chemical Engineering, Sun Yat-Sen University, Guangzhou 510275, China.

*E-mail: zhangjp7@mail.sysu.edu.cn

Supplementary Index

Experimental details.

Scheme S1. Representation of the column breakthrough experiment.

Figure S1. PXRD patterns of **1** and **1a** after heating treatments.

Figure S2. Thermogravimetry-mass curve of **1a**.

Figure S3. N_2 sorption isotherms of **1** and **1a** measured at 77 K.

Figure S4. Time-dependent CO_2 adsorption for **1a** under air atmosphere.

Figure S5. The obtained Virial and Langmuir-Freundlich fitting parameters of **1a**.

Figure S6. Linear fitting of the van't Hoff isochore at different CO_2 adsorption amounts measured at 298, 313 and 328 K.

Figure S7. IR spectra of **1a** with and without adsorbed CO_2.

Figure S8. Heat flows of **1a** determined *via* DSC.

Figure S9. Breakthrough curves of Fig. 5 expressed using specific injection amount, specific breakthrough time and breakthrough time as abscissa.

Table S1. The pK_b values of the diamine.

Table S2. The noteworthy CO_2 adsorption performances of PCPs at 298 K.

Table S3. The noteworthy CO_2 adsorption performances of PCPs at 328 K.

Table S4. Comparing the performance of capturing CO_2 from simulated flue gas of **1a** with the highest reported values.

Table S5. Comparing the performances of capturing CO_2 from simulated flue gas through a single breakthrough operation of **1a** with the highest reported values.

Experiment Section

Materials and General Methods. Reagents and solvents were commercially available and were used without further purification. All anhydrous solvents were dried using 3A molecular sieve. Elemental analyses (C, H, N) were performed with a Vario EL elemental analyzer. Thermogravimetry analysis was performed using a NETZSCH (TG 209 F3 Tarsus) system. Ultrahigh-purity-grade (99.999%) N_2 and CO_2 were used for the TVSA and TSA process. PXRD patterns were collected on a Bruker D8 Advance diffractometer (Cu Kα) at room temperature. Diffused Reflection Fourier Transform Infrared Spectra (DR-FTIR) were measured by a Bruker VERTEX 70 spectrometer in the 400–4000 cm^{-1} region.

Synthesis of $[Mg_2(dobdc)]$ (**1**). **1** was synthesized according to the literature method. A mixture of $Mg(NO_3)_2{\cdot}6H_2O$ (0.475 g, 1.85 mmol), H_4dobdc (0.111 g, 0.559 mmol, Aldrich), 15:1:1 (*v*/*v*/*v*) mixture of DMF–ethanol–water (50 mL) was stirred for 10 min in air, then transferred and sealed in a 100-mL Teflon reactor, which was heated in an oven at 125 °C for 24 h. The oven was cooled to room temperature at a rate of 5 °C h^{-1}. The obtained mixture was filtered, successively washed by DMF and MeOH twice, soaked in MeOH for four days, filtered, and finally heated at 250 °C over 5 h to give light yellow microcrystalline powder, yielding the dark yellow microcrystalline material. The yield of the reaction, determined from the weight of the solvent-free material $[Mg_2(dobdc)]$, is 93% based on H_4dobdc. Anal. Calcd (%) for $[Mg_2(H_2O)_2(dobdc)]{\cdot}6.2H_2O$ ($C_8H_{18.4}Mg_2O_{14.2}$): C, 24.61; H, 4.75. Found: C, 24.57; H, 4.61.

Synthesis of $[Mg_2(dobdc)(N_2H_4)_{1.8}]$ (**1a**). A sample of fully activated **1** (100 mg, 0.31 mmol) was loaded in a Schlenk flask, to which a solution of toluene (100 mL) with anhydrous N_2H_4 (N_2H_4, 0.42 mL, 6.27 mmol) was added. The suspension was stirred for 48 h under N_2 atmosphere at room temperature. The solid was separated by centrifugation and washed with toluene several times. The resulting residue was successively soaked in hexane for 72 h and anhydrous methanol for 24 h, filtered, and finally evacuated at 130 °C for 12 h to obtain an light yellow powder (Yield: 98%). Anal. Calcd (%) for $[Mg_2(dobdc)(N_2H_4)_{1.8}]{\cdot}2(CH_3OH)$ ($C_{10}H_{17.6}N_{3.6}Mg_2O_{8.2}$): C, 32.63; H, 4.82; N, 13.70. Found: C, 32.39; H, 4.95; N, 13.46.

Gas Sorption Measurements. The sorption isotherms were measured with an automatic volumetric adsorption apparatus (BELSORP-max). The as-synthesized sample (weight of about 200~300 mg) was placed in the sample tube and dried for 12 h at 130 °C to remove the remnant solvent molecules prior to measurements. CO_2 (99.999%) and N_2 (99.999%) were used for all measurements. The temperatures were controlled by a liquid-nitrogen bath (77 K) or a water bath (298, 313 and 328 K). For the CO_2 sorption measurement at 413 K, an oil bath (IKA ETS-D5) normally used for chemical synthesis, was used to control the measurement temperature of the high-temperature isotherm. During the measurement process, the ascending and descending of oil bath was controlled manually. It should be noted that the manual operation must be very careful to avoid damage to the gas adsorption apparatus. With the isotherms at 298, 313 and 328 K, as well as the Q_{st} profile (direct calculation by the Clausius-Clapeyron equation without data fitting), the uptake at 413 K and 1.0 bar can be predicted to about 1.84 mmol g^{-1}, which is higher than the value obtained from the adsorption isotherm measured at 413 K (Fig. 2a). This phenomenon is ascribed to the large temperature interval of 115 K (the Clausius-Clapeyron equation is not completely linear), which is similar with the result reported in the literature (*Chem. Sci.*, **2015**, *6*, 3697–3705).

Theoretical calculations. All of the calculations were completed in the Materials Studio 5.5 package. The N_2H_4-grafted structures were optimized by molecular mechanics (MM) based on the universal force field (UFF), during which the unit cell parameters and the positions of all of the metal atoms were fixed. The Ewald summation method was used for both the

electrostatic and van der Waals terms, and the buffer width was set to 0.5 Å. To further obtain more accurate information for the hydrogen bonds between pairs of adjacent amine groups, the structures obtained from MM simulation was further optimized by periodic density functional theory (PDFT) method by the Dmol^3 module. The widely used generalized gradient approximation (GGA) with the Perdew-Burke-Ernzerhof (PBE) functional and the double numerical plus d-functions (DNP) basis set, TS for DFT-D correction as well as the Effective Core Potentials (ECP) were used. The energy, force and displacement convergence criterions were set as 1×10^{-5} Ha, 2×10^{-3} Ha and 5×10^{-3} Å, respectively.

X-ray Crystallography. The PXRD patterns were collected (0.02 °/step, 10 seconds/step) on a Bruker D8 Advance diffractometer (Cu Kα) at room temperature. Pawley refinements were performed by the Reflex plus module of Material Studio 5.0. Pawley refinements were performed in the 2θ range of 5–60° on unit-cell parameters, zero point and background terms with Pseudo-Voigt profile function and Berar-Baldinozzi asymmetry correction function under the *R*-3 space group, yielding the following parameters: a = 25.87(2) Å, c = 6.800(4) Å, V = 3941(8) Å^3, R_p = 3.90%, R_{wp} = 4.72% for **1** and a = 25.92(1) Å, c = 6.817(3) Å, V = 3966(4) Å^3, R_p = 3.17%, R_{wp} = 4.18% for **1a** (Fig. S1).

CO_2/N_2 breakthrough curve measurements. The column with a length of 10 cm and an internal diameter of 0.46 cm (V = 1.66 cm^3) contained 1.076 g of **1a** (apparent density: 0.65 g cm^{-3}, column voidage: 0.448) for the experiment using 10:90 CO_2/N_2 mixture. For the 1:999 CO_2/N_2 mixture, 0.134 g of **1a** was packed into a stainless-steel column (5 cm length × 0.46 cm internal diameter, V = 0.83 cm^3, apparent density: 0.61 g cm^{-3}, column voidage: 0.482) and the remaining volume in the column was filled by glass wool (0.61 cm^3, void: 0.27 cm^3). Since the CO_2 concentration is very low, the experiments need very long time to reach the breakthrough point. Therefore, a smaller sample amount was used for the 1:999 CO_2/N_2 breakthrough experiment. The column was connected to the injection and sampling ports by stainless-steel pipes with a combined length of 40 cm and an internal diameter of 0.30 cm (V_{tube} = 0.150×0.150×3.14×40 = 2.84 cm^3). The column and most parts of the pipelines between the injection and sampling ports were placed in a temperature-controlling oven (Scheme S1). The flow rates (mL min^{-1} at 298.15 K and 101.325 kPa) of pure gases were regulated by mass flow controllers. Before breakthrough experiments, the columns were activated by passing He (10 mL min^{-1}) and heated at 403 K for 10 hours, and then cooled to the measurement temperature of 313(1) K. Pure N_2 (1.8 mL min^{-1}) and CO_2 (0.2 mL min^{-1}) or pure N_2 (4.5 mL min^{-1}) and mixed standard gas 1:99 CO_2/N_2 (*v*/*v*) (0.5 mL min^{-1}) were mixed to generate the dry (0% RH) 10:90 and 1:999 CO_2/N_2 mixtures, respectively. Passing the dry gas mixtures through a water vapor saturator produced the wet gas mixture for the injection port. The temperature and relative humidity (RH) were measured by an Aosong AS108R digital temperature-humidity sensor. Prior to the breakthrough measurements in the presence of high humidity, wet He with the same RH was introduced to the adsorbent bed until water saturation was detected. Then the wet He was switched to wet CO_2/N_2 mixture to start the breakthrough experiment. The temperature and relative humidity was measured by a digital temperature-humidity sensor. The gas stream at the outlet of the column was analyzed on line by using a chromatographic analyzer (Agilent 7890A) with a TCD detector (G3440A) and a PLOT/Q column.

The amount of CO_2 (q) retained by the column at a particular specific injection amount τ, can be calculated by

$$q(\tau) = 0.1*(\tau - \int_0^\tau f(\tau)\mathrm{d}\tau)$$

for the 10:90 CO_2/N_2 mixture, or

$$q(\tau) = 0.001*(\tau - \int_0^\tau f(\tau)\mathrm{d}\tau)$$

for the 1:999 CO_2/N_2 mixture

where the breakthrough curve is expressed by the function $f(\tau)$. $q(\tau)$ reaches a maximum/saturation value q_{max} after the outlet CO_2 concentration stabilizes at the value of the inlet one.

The CO_2 adsorbed by the adsorbent in the column (Q) can be calculated by

$$Q = q_{\max} - q_c$$

where the q_c is the CO_2 residing in the free space of the column and the pipeline, which is (1.66×0.448+2.84)×0.1×273/313/22.4/1.076 = 0.0130 and (0.830×0.482+0.27+2.84)×0.001×273/313/22.4/0.134 = 0.00102 mmol g^{-1} for the 10:90 and 1:999 CO_2/N_2 experiments, respectively.

Calculation of approximate regeneration energy. The specific heat (2.01 J g^{-1} K^{-1}) of **1a** was measured by DSC. The sensible heat required for regeneration (200 J g^{-1}) is the specific heat (2.0 J g^{-1} K^{-1}) multiplied by the temperature change (100 K, see Fig. S6). The working capacity was obtained as 3.52 mmol g^{-1} between 15:85 CO_2/N_2 (v/v) mixture at 313 K and a pure CO_2 flow at 413 K. To remove this CO_2, approximately 268 J g^{-1} (obtaining via the integration of the coverage-dependent CO_2 adsorption enthalpy curve obtained by the Clausius-Clapeyron equation (original isotherms without fitting)) are required. To adsorb 1 kg (22.72 mol) of CO_2, 6.45 kg of **1a** are necessary. Thus, the regeneration energy is:

6.45 kg* (200 kJ kg^{-1}+ 268 kJ kg^{-1}) = 3.02 MJ kg^{-1} CO_2.

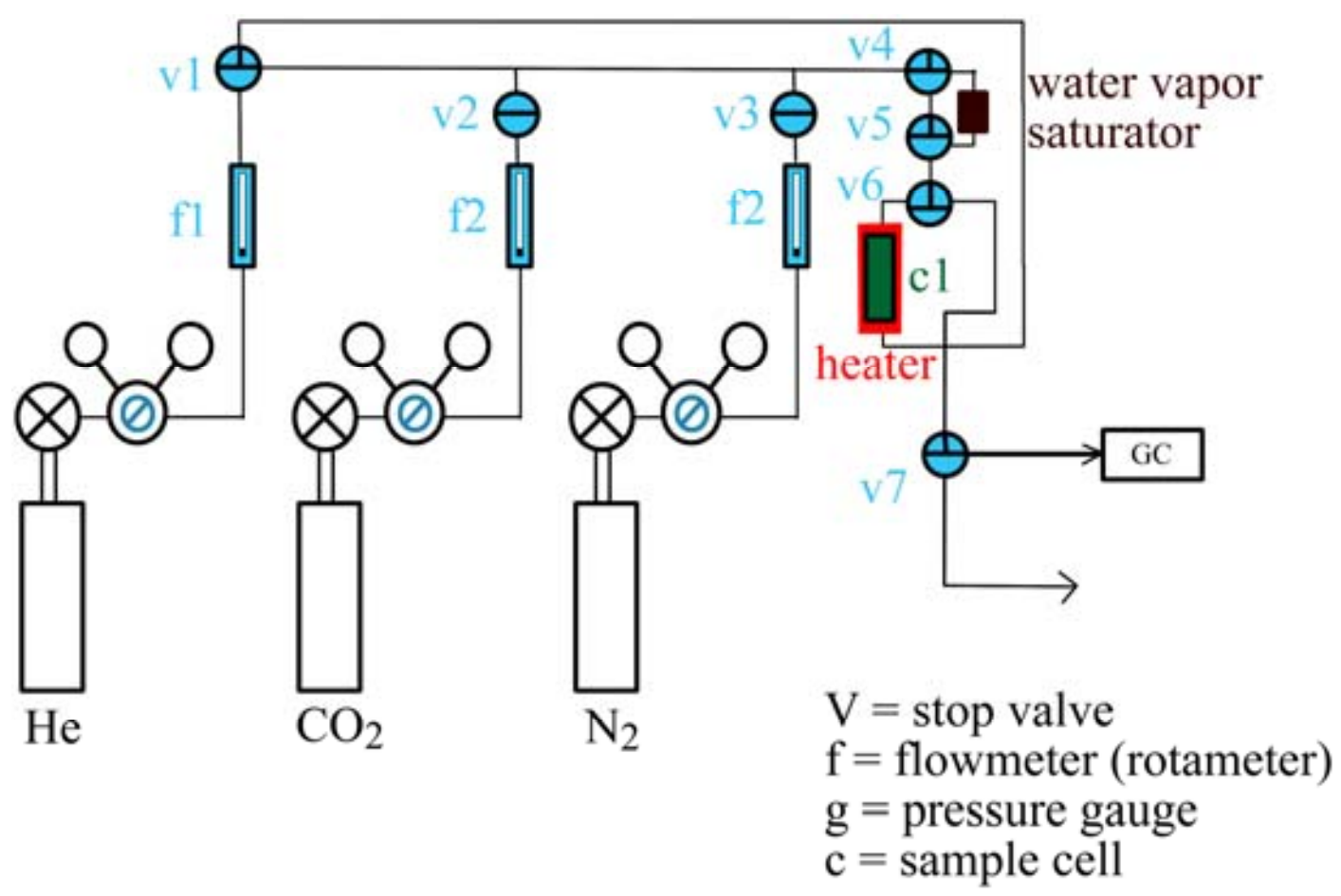

Scheme S1. Representation of the column breakthrough experiment.

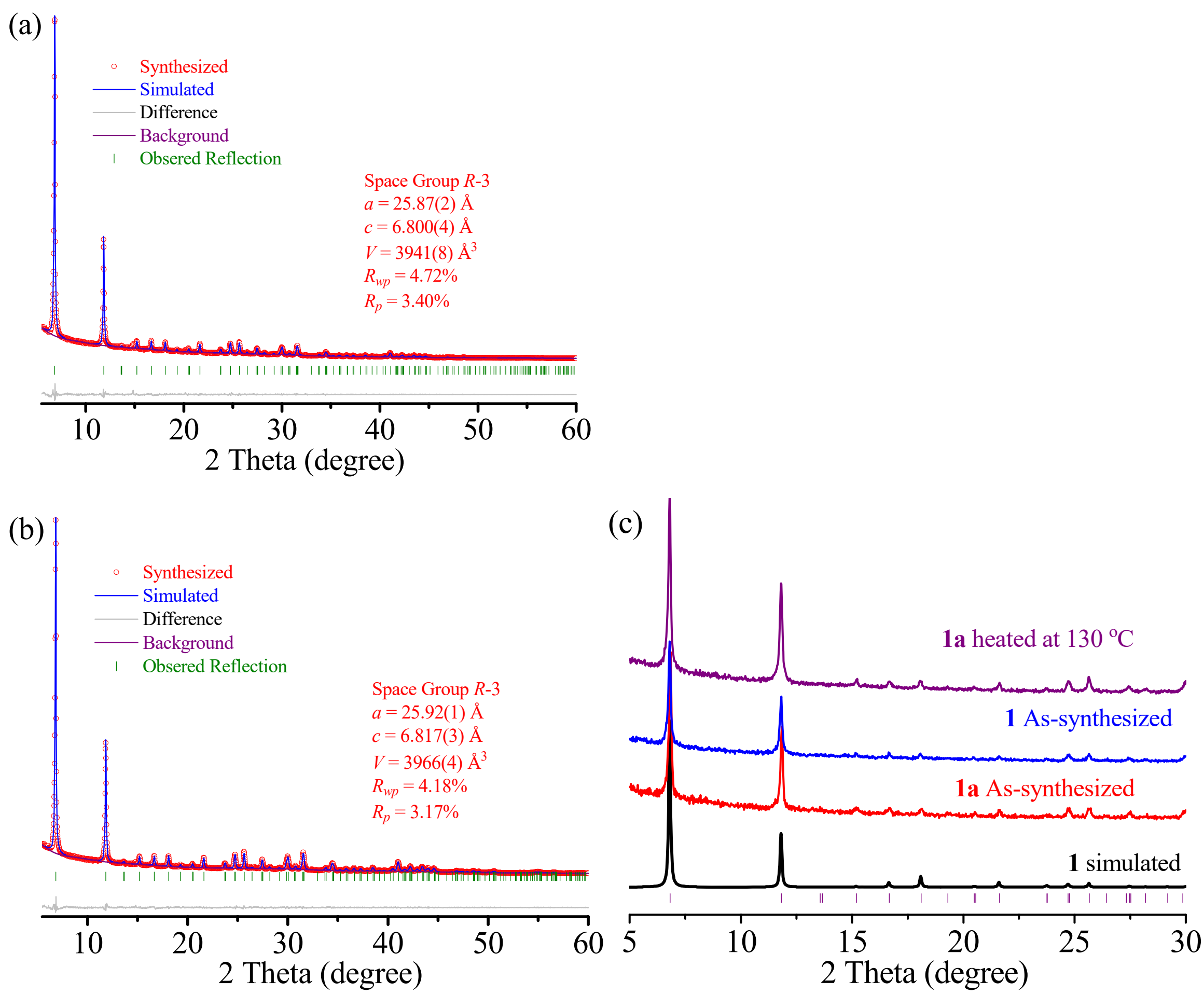

Figure S1. PXRD patterns of **1** and **1a** after heating treatments.

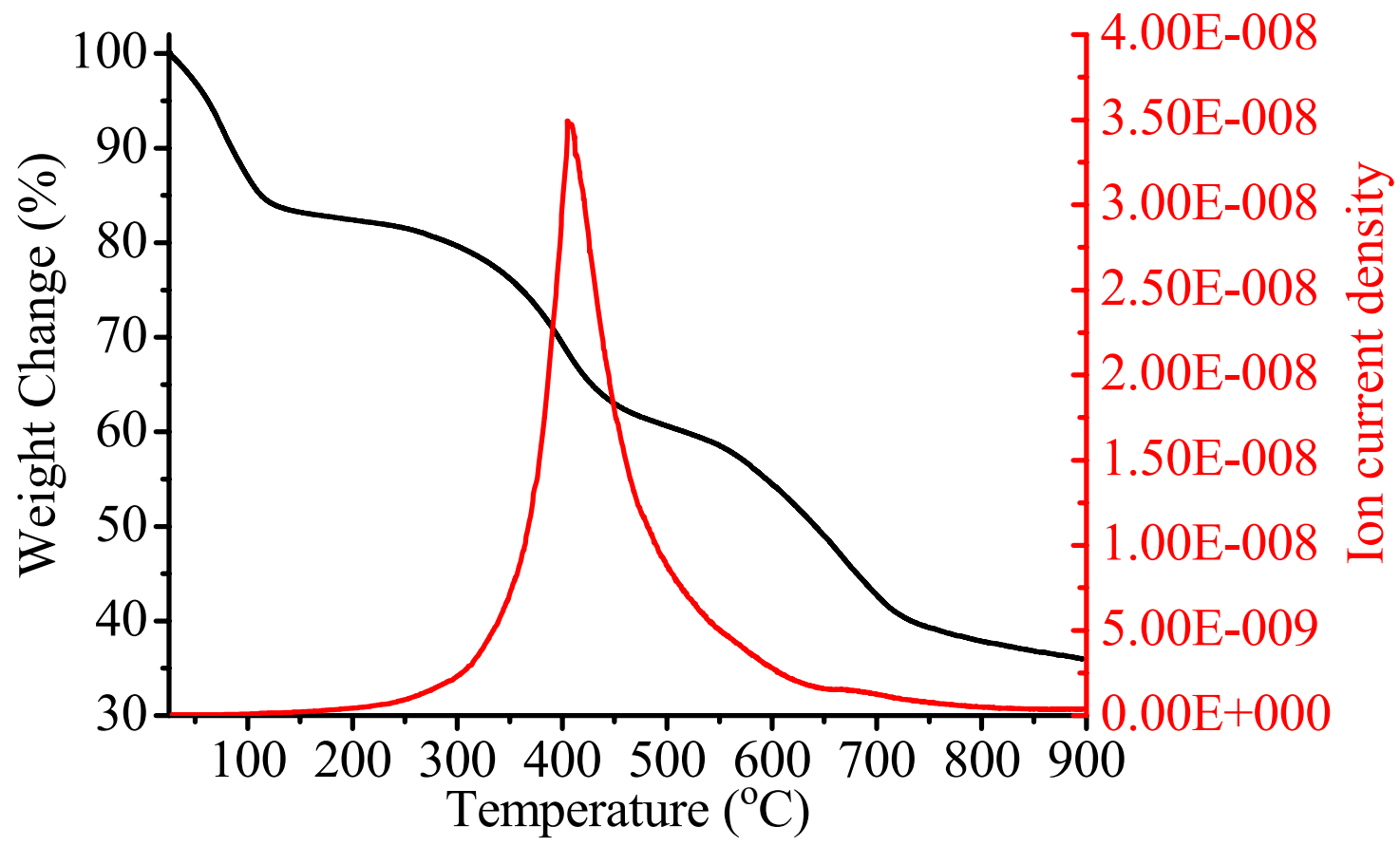

Figure S2. Thermogravimetry-mass curve of **1a** under N_2 with temperature increased with 5 °C min^{-1}.

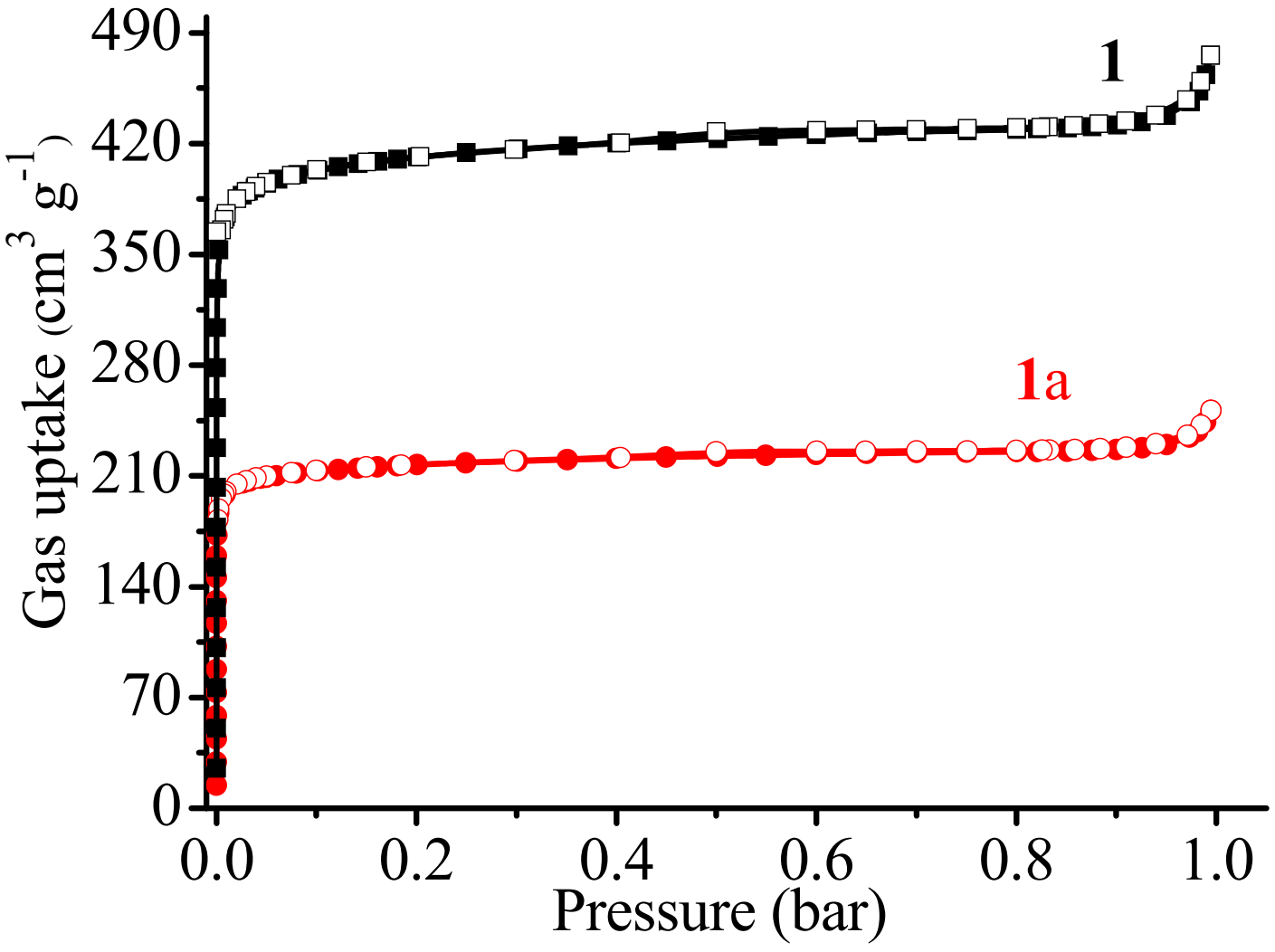

Figure S3. N_2 adsorption (filled) and desorption (open) isotherms of **1** and **1a** measured at 77 K.

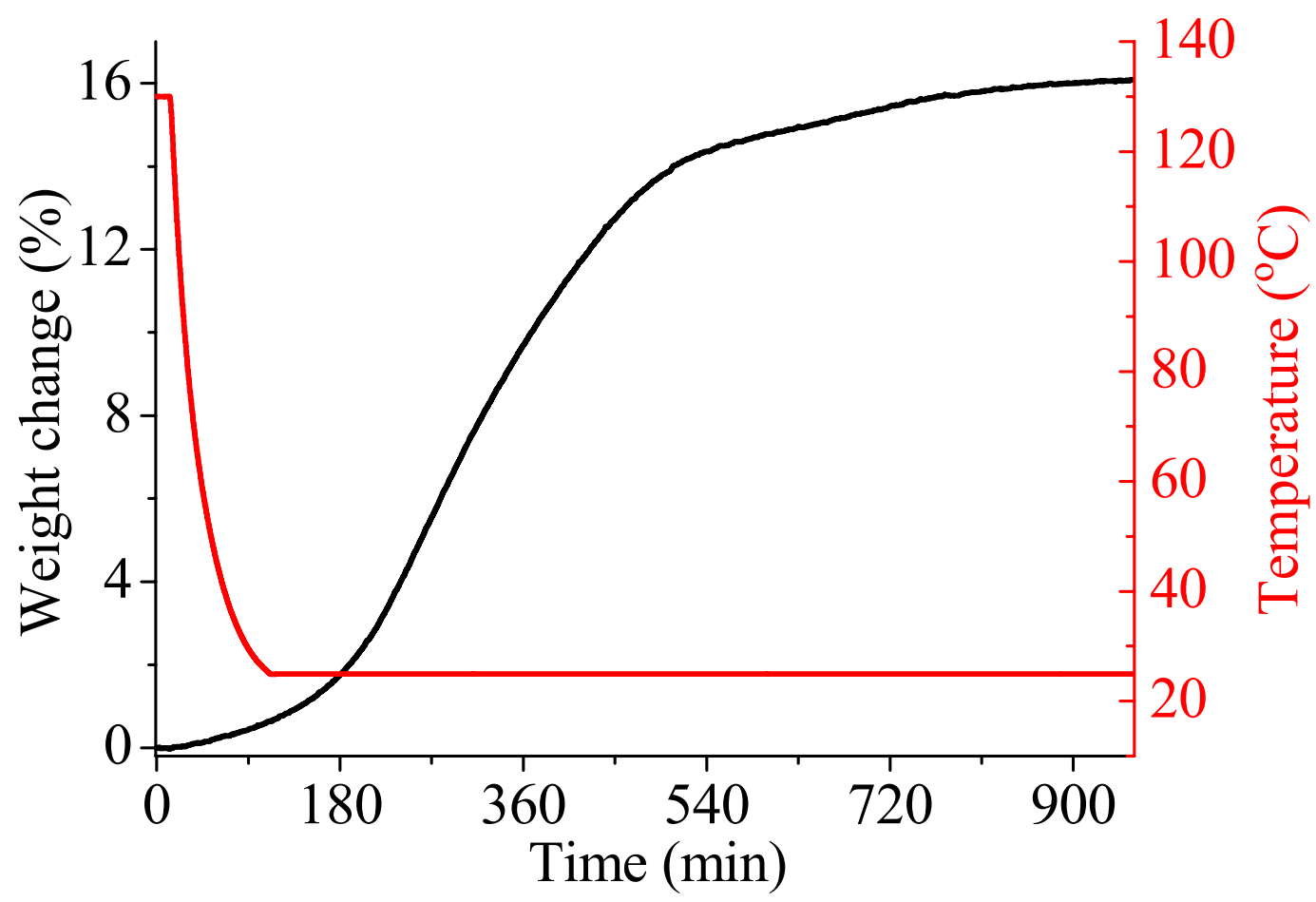

Figure S4. Time-dependent CO_2 adsorption for **1a** under air atmosphere.

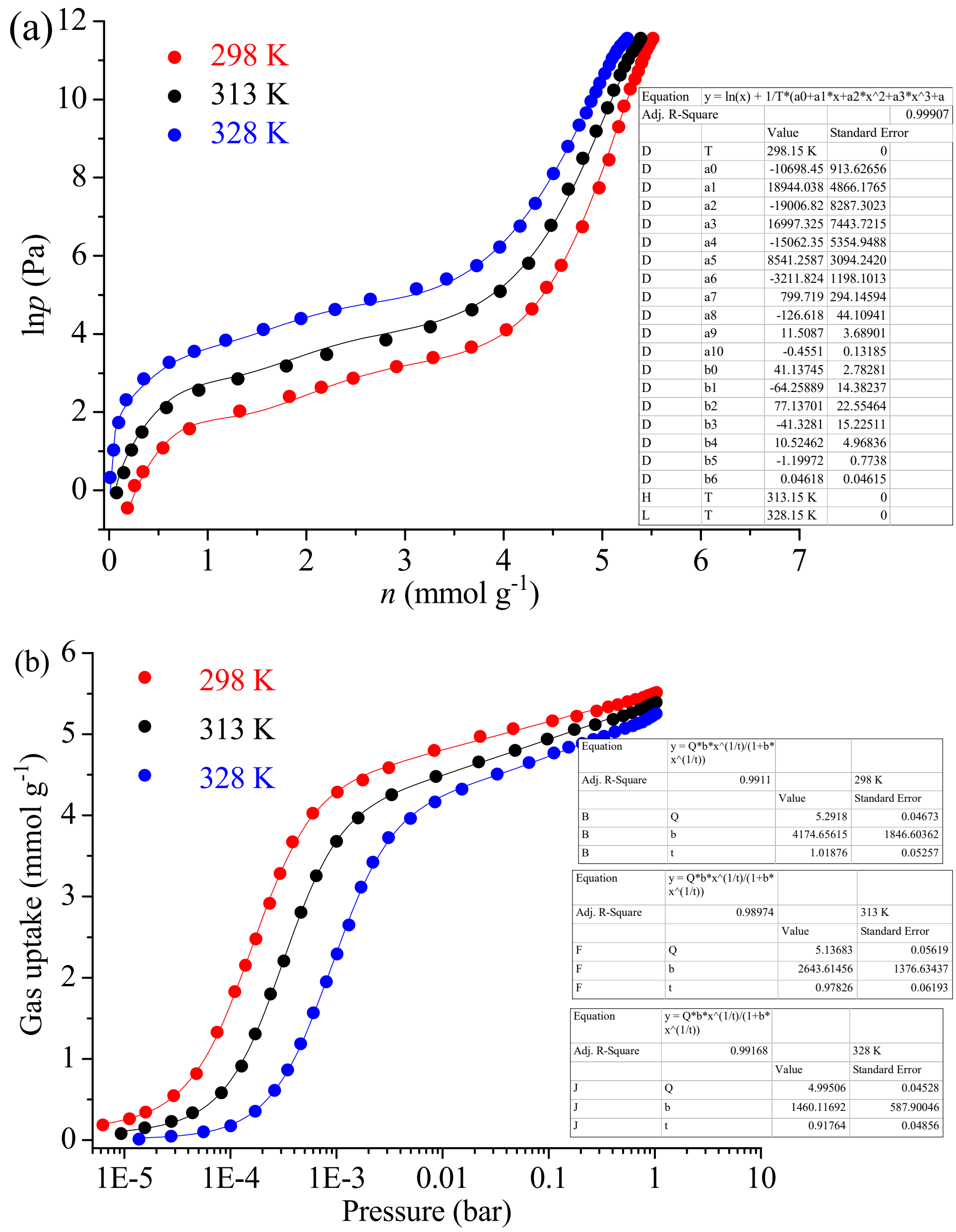

Figure S5. (a) Virial and (b) Langmuir-Freundlich fittings (lines) of the CO_2 adsorption isotherms (points) of **1a** measured at 298, 313 and 328 K.

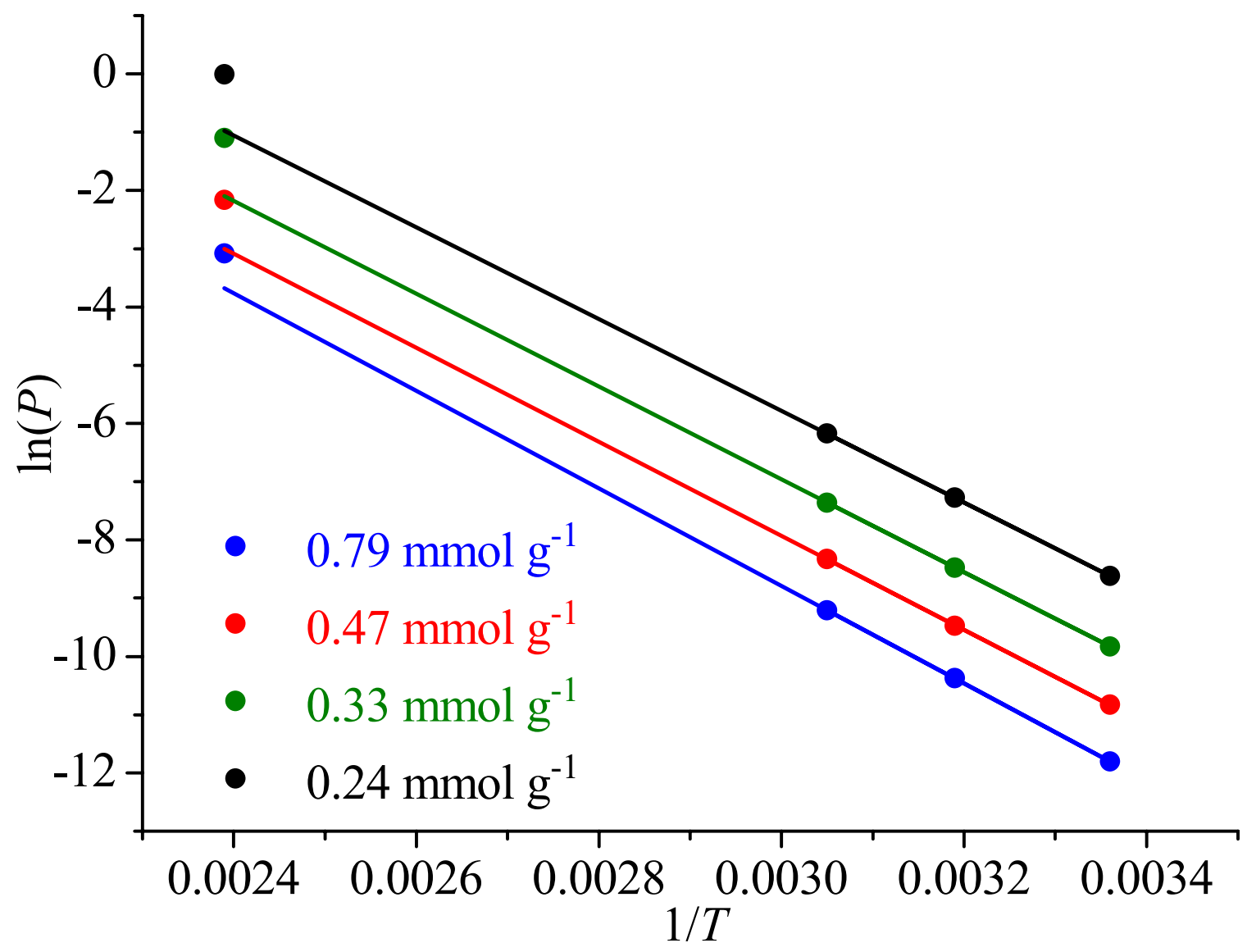

Figure S6. Linear fitting of the van't Hoff isochore at different CO_2 adsorption amounts measured at 298, 313 and 328 K. Reaction enthalpies are not constants, but usually vary little against temperature and treated as constants within some temperature ranges. Note that the structures of metal-organic frameworks are relatively sensitive to temperature, meaning that their adsorption enthalpies can have relatively large variations against temperature. In other words, van't Hoff isochores are always not strictly linear, but can be regarded as linear within some temperature ranges dependent on the temperature sensitivity of the reaction system. There is large temperature interval between 298 and 413 K, thus the data at 413 K deviate somewhat from the predictions using the linear van't Hoff isochores.

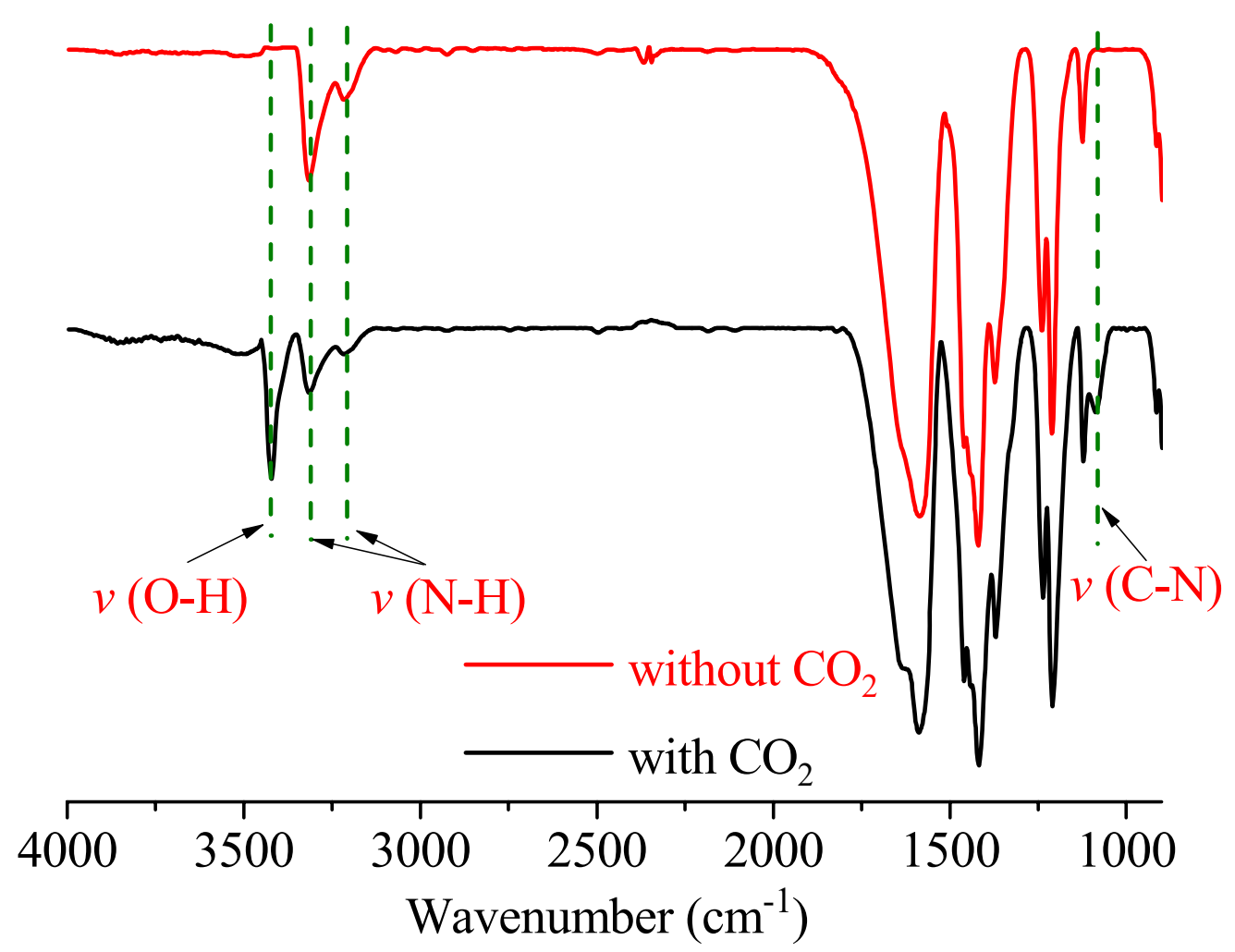

Figure S7. *In situ* IR spectra of **1a** with and without adsorbed CO_2 measured at 313 K.

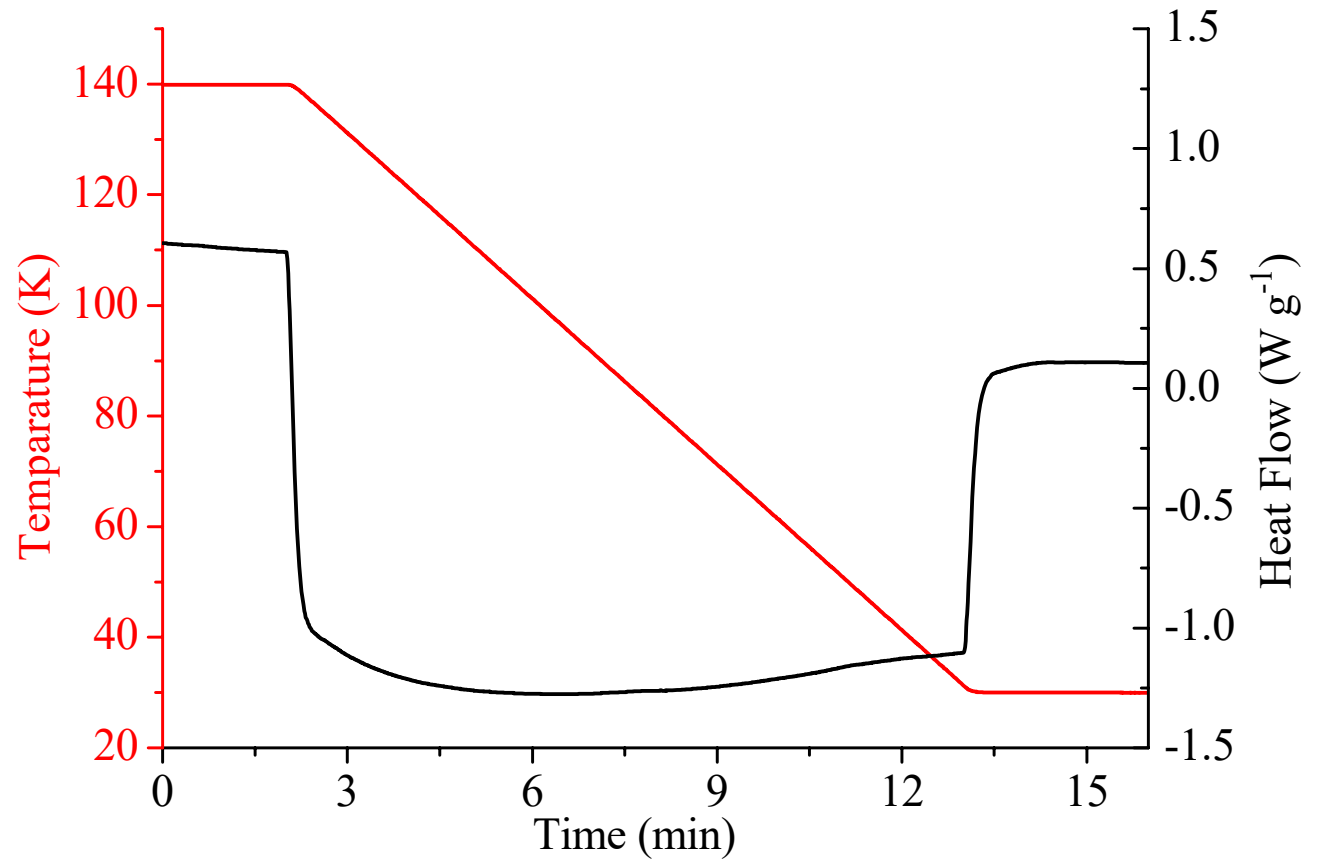

Figure S8. Heat flows from **1a**, as determined *via* DSC.

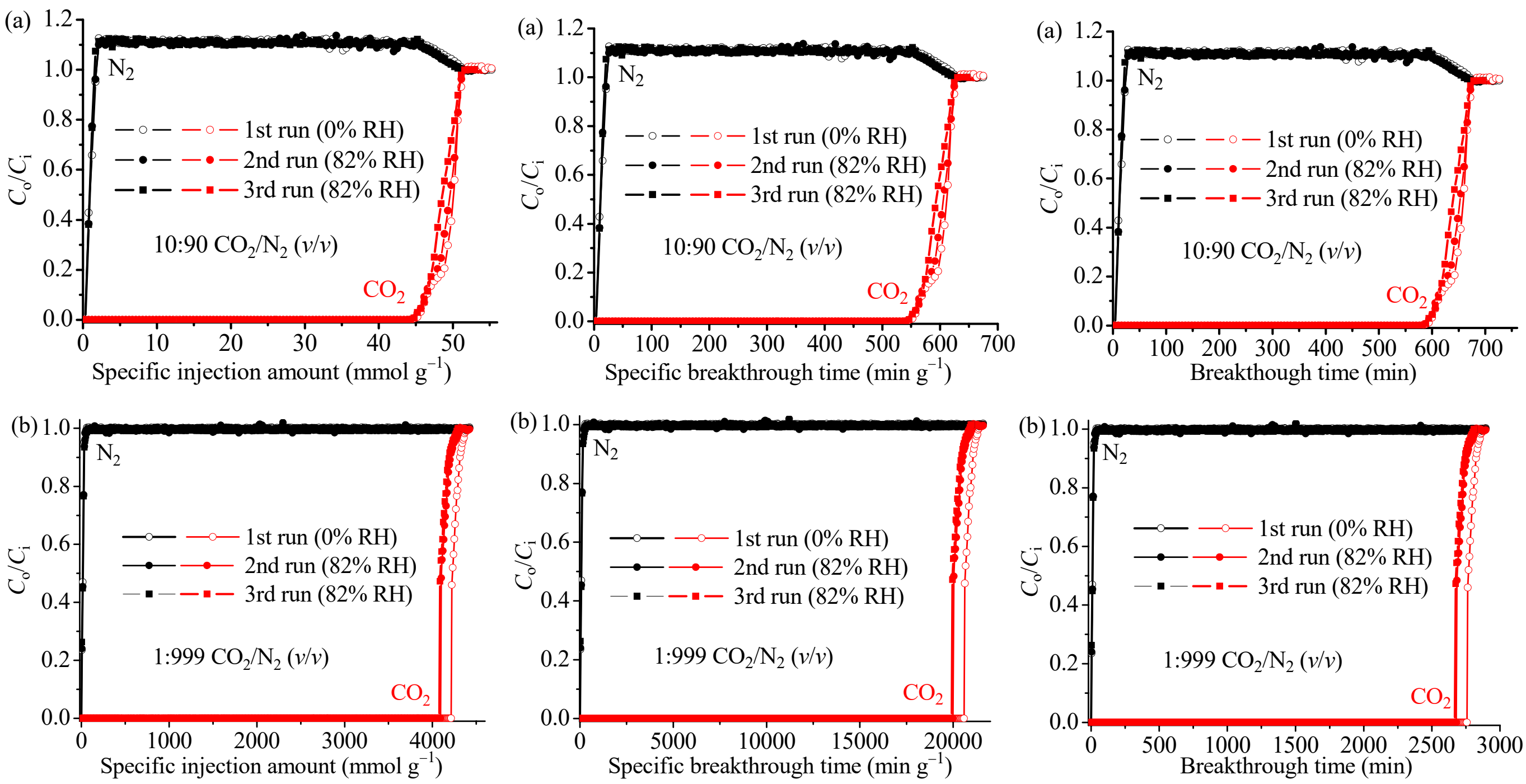

Figure S9. Breakthrough curves of Fig. 5 expressed using specific injection amount (mmol g^{-1}, left), specific breakthrough time (min g^{-1}, middle) and breakthrough time (min, right) as abscissa, respectively.

Table S1. The pK_b values of the diamine.

	pK_b
N,*N*'-dimethylethylenediamine	3.84
Ethylenediamine	4.02
Hydrazine	5.93
Ammonia	4.79

Table S2. Comparison of the noteworthy CO_2 adsorption performances of PCPs at 298 K. The highest and the second highest values were highlighted in blue and green, respectively.

Compound (common name)	Q_{st} (kJ mol^{-1})	D_c (g cm^{-3})	CO_2 uptake (mmol g^{-1})			CO_2 uptake (mmol cm^{-3})			Type of active sites	Ref
			0.4 mbar	0.15 bar	1 bar	0.4 mbar	0.15 bar	1 bar		
**1a**	90[a], 90[b], 118[d]	1.178	3.89	5.18	5.51	4.58	6.10	6.49	LBS+OMS	This work
**1**[g]	47[c]	0.920	0.088	5.71	8.04	0.104	5.25	7.40	OMS	S1
eda-Mg_2(dobpdc)	51[e]	0.955	2.85	3.49	4.56	2.72	3.33	4.36	LBS+OMS	S2
mmeda-Mg_2(dobpdc)	71[f]	1.073	2.10	3.15	3.86	2.25	3.38	4.14	LBS+OMS	S3
SIFSIX-3-Cu	54	1.60	1.24	2.51	2.58	1.98	4.02	4.13	NA	S4
SIFSIX-3-Zn	45	1.62	0.13	2.41	2.51	0.21	3.90	4.07	NA	S4
mmeda-CuBTTri	66[a], 96[f]	1.059	NA	2.12	4.2	NA	2.24	4.45	LBS+OMS	S5
SIFSIX-2-Cu-i	31.9	1.246	0.0684	2.25	5.41	0.085	2.80	6.74	NA	S6
[Mn_2Cl_2(bbta)(OH)]	99[a]	1.227	0.95	4.01	7.14	1.16	4.92	8.76	LBS+OMS	S7
[Co_2Cl_2(bbta)(OH)]	110[a]	1.354	1.13	4.10	6.70	1.53	5.55	9.07	LBS+OMS	S7
dmeda-Mg_2(dobpdc)	75[e]	1.071	NA	3.75	4.95	NA	4.02	5.30	LBS+OMS	S11

mmeda = *N*,*N*'-dimethylethylenediamine; BTTri = 1,3,5-tris(1H-1,2,3,-triazol-5-yl)benzene; dobpdc = 4,4'-dioxido-3,3'-biphenyldicarboxylate; dobdc = 2,5-dioxido-1,4-benzenedicarboxylate; SIFSIX = SiF_6^{2-} anions; PEI = polyethyleneimine; dmeda = *N*,*N*-dimethylethylenediamine; LBS = Lewis basic site.

[a] Obtained by the Virial fitting method.

[b] Obtained by the Clausius-Clapeyron equation (original isotherms without fitting).

[c] Obtained by the Clausius−Clapeyron equation and isotherms fitted by the Toth model.

[d] Obtained by the Clausius−Clapeyron equation and isotherms fitted by the Langmuir−Freundlich model.

[e] Obtained by the Clausius−Clapeyron equation and isotherms fitted by the dual-site Langmuir−Freundlich model.

[f] Obtained by the Clausius−Clapeyron equation and isotherms fitted by the dual-site Langmuir model.

[g] 296 K.

NA = Not Applicable or Not Available.

Table S3. Comparison of the noteworthy CO_2 adsorption performances of PCPs at 328 K. The highest and the second highest values were highlighted in blue and green, respectively.

Compound (common name)	Q_{st} (kJ mol^{-1})	D_c (g cm^{-3})	CO_2 uptake (mmol g^{-1})			CO_2 uptake (mmol cm^{-3})			Type of active sites	Ref
			0.4 mbar	0.15 bar	1 bar	0.4 mbar	0.15 bar	1 bar		
**1a**	90^a, 90^b, 118^d	1.178	1.02	4.84	5.24	1.22	5.70	6.17	LBS+OMS	This work
**1**	47^c	0.920	0.041	4.19	7.01	0.038	3.85	6.45	OMS	S1
eda-Mg_2(dobpdc)^g	51^e	0.955	0.12	3.49	4.25	0.11	3.33	4.06	LBS+OMS	S2
mmeda-Mg_2(dobpdc)^g	71^f	1.073	0.12	2.71	3.13	0.11	2.91	3.36	LBS+OMS	S3
SIFSIX-3-Cu	54	1.60	0.24	2.29	2.37	0.38	3.66	3.79	NA	S4
SIFSIX-3-Zn	45	1.62	0.029	2.25	2.51	0.047	3.64	4.07	NA	S4
mmeda-CuBTTri	66^a, 96^f	1.059	NA	2.55	4.08	NA	2.70	4.32	LBS+OMS	S5
SIFSIX-2-Cu-i	31.9	1.246	NA	2.46	5.48	NA	3.06	6.83	NA	S6
[Mn_2Cl_2(bbta)(OH)]	99^a	1.227	0.179	2.34	5.06	0.219	2.87	6.21	LBS+OMS	S7
[Co_2Cl_2(bbta)(OH)]	110^a	1.354	0.194	2.38	5.10	0.263	3.22	6.90	LBS+OMS	S7
dmeda-Mg_2(dobpdc)^g	75^e	1.071	NA	NA	3.78	NA	NA	4.05	LBS+OMS	S11

mmeda = *N*,*N*'-dimethylethylenediamine; BTTri = 1,3,5-tris(1*H*-1,2,3,-triazol-5-yl)benzene; dobpdc = 4,4'-dioxido-3,3'-biphenyldicarboxylate; dobdc = 2,5-dioxido-1,4-benzenedicarboxylate; SIFSIX = SiF_6^{2-} anions; PEI = polyethyleneimine; dmeda = *N*,*N*-dimethylethylenediamine; LBS = Lewis basic site.

[a] Obtained by the Virial fitting method.

[b] Obtained by the Clausius-Clapeyron equation (original isotherms without fitting).

[c] Obtained by the Clausius−Clapeyron equation and isotherms fitted by the Toth model.

[d] Obtained by the Clausius−Clapeyron equation and isotherms fitted by the Langmuir−Freundlich model.

[e] Obtained by the Clausius−Clapeyron equation and isotherms fitted by the dual-site Langmuir−Freundlich model.

[f] Obtained by the Clausius−Clapeyron equation and isotherms fitted by the dual-site Langmuir model.

[g] 323 K.

NA = Not Applicable or Not Available.

Table S4. Comparing the performances of capturing CO_2 from simulated flue gas (T = 313 K, 15:85 CO_2/N_2 mixture) of **1a** with the highest reported values (measured by thermogravimetry).

Compound	Density ($g\ cm^{-3}$)	The smallest aperture size (Å)	Uptake from single-component isotherm (wt%)	Gravimetric uptake difference (wt%) / Adsorption + desorption time (min)	Volumetric uptake difference ($mmol\ cm^{-3}$) / Adsorption + desorption time (min)	Regeneration condition	Ref
**1a**	1.178	4.8	21.8	19.0 / 40 + 43<br>17.1 / 17 + 43<br>11.4 / 13 + 43	5.01 / 40 + 43<br>4.58 / 17 + 43<br>3.05 / 13 + 43	N_2 purge at 403 K	This work
				15.5 / 48 + 54<br>13.6 / 22 + 54<br>8.02 / 12 + 54	4.15 / 48 + 54<br>3.64 / 22 + 54<br>2.15 / 12 + 54	CO_2 purge at 413 K	
eda-Mg_2(dobpdc)	0.955	11	15.4	14.6 / 90 + 100<br>8.73 / 30 + 100	3.17 / 90 + 100<br>1.89 / 30 + 100	Ar purge at 423 K	S2
mmeda-Mg_2(dobpdc)	1.073	11	13.2	11.1 / 18 + 25	2.71 / 18 + 25	N_2 purge at 393 K	S3
				8.03 / 22 + 25	1.95 / 22 + 25	CO_2 purge at 423 K	
[Mn_2Cl_2(bbta)(OH)]	1.227	7.6	13.3	13.1 / 36 + 39	3.65 / 36 + 39	N_2 purge at 358 K	S7
[Co_2Cl_2(bbta)(OH)]	1.354	7.4	13.8	13.4 / 37 + 19	4.12 / 37 + 19	N_2 purge at 358 K	
				9.20 / 42 + 24	2.83 / 42 + 24	CO_2 purge at 358 K	
dmeda-Mg_2(dobpdc)	1.071	11	13.6	13.5 / 100 + 100	3.29 / 100 + 100	Ar purge at 403 K	S11
				13.5 /120 + 150	3.29 / 120 + 150	CO_2 purge at 403 K	

Table S5. Comparing the performances of capturing CO_2 from simulated flue gas (10:90 CO_2/N_2 (*v*/*v*)) through a single breakthrough operation of **1a** with the highest reported values. The highest and the second highest values were highlighted in blue and green, respectively.

Compound	Density (g cm^{-3})	CO_2 breakthrough point (mmol g^{-1} / mmol cm^{-3})	Single-component CO_2 isotherm uptake at 0.1 bar (mmol g^{-1} / mmol cm^{-3})	Breakthrough CO_2 uptake (mmol g^{-1} / mmol cm^{-3})	Breakthrough /Isotherm uptake ratio (%)	Relative humidity (%)	Breakthrough CO_2 uptake ratio between humid and dry conditions (%)	Ref
**1a**[a, f]	1.178	44.3 / 52.2	4.97 / 5.85	4.89 / 5.76	98	82(3)	98	This work
**1**[b]	0.92	42.2 / 38.8	4.93 / 4.54	4.24 / 3.90	86	90	35	S8
SGU-29[b]	1.97	25.5 / 50.2	2.76 / 5.44	2.65 / 5.22	96	90	100	S8
SIFSIX-3-Cu[b]	1.58	20.3 / 32.1	2.54 / 4.02	2.34 / 3.70	92	90	93	S8
SIFSIX-3-Zn[b]	1.57	NA	2.27 / 3.57	1.59 / 2.50	70	90	96	S8
AM-6[b]	2.01	21.7 / 43.7	2.44 / 4.91	2.24 / 4.52	92	90	100	S8
ETS-10[b]	1.93	19.0 / 36.6	2.04 / 3.93	1.86 / 3.59	91	90	96	S8
UTSA-16[b]	1.66	NA	1.67 / 2.77	1.08 / 1.80	65	90	90	S8
ETS-4[b]	2.20	17.6 / 38.8	2.03 / 4.46	1.83 / 4.01	90	90	95	S8
NaX[b]	1.42	19.8 / 28.1	2.42 / 3.43	2.01 / 2.85	83	90	81	S8
[Ni_2(dobdc)][b]	1.19	NA	3.00 / 3.57	2.61 / 3.10	87	90	78	S8
mmeda-Mg_2(dobpdc)[b]	0.955	NA	3.69 / 3.52	2.84 / 2.71[c]	77[c]	48	NA	S9
MAF-X27ox[a, f]	1.354	22.4 / 30.3	2.60 / 3.52	2.49 / 3.37	96	82(3)	100	S7
ZIF-300[b, f]	1.351	NA	NA	NA	NA	80	98	S10

Amount of purified N_2 is obtained by integration of the N_2 breakthrough curve to the CO_2 breakthrough point;

[a] Measured at 313 K;

[b] Measured at 298 K;

[c] Measured under humid condition;

[f] With pre-equilibration of the bed under a humid inert gas stream;

NA = Not Applicable or Not Available.

References

(S1) S. R. Caskey, A. G. Wong-Foy, A. J. Matzger, *J. Am. Chem. Soc.* **2008**, *130*, 10870.

(S2) W. R. Lee, S. Y. Hwang, D. W. Ryu, K. S. Lim, S. S. Han, D. Moon, J. Choi, C. S. Hong, *Energy Environ. Sci.* **2014**, *7*, 744.

(S3) T. M. McDonald, W. R. Lee, J. A. Mason, B. M. Wiers, C. S. Hong, J. R. Long, *J. Am. Chem. Soc.* **2012**, *134*, 7056.

(S4) O. abilityShekhah, Y. Belmabkhout, Z. Chen, V. Guillerm, A. Cairns, K. Adil and M. Eddaoudi, *Nat. Commun.*, 2014, **5**, 4228.

(S5) T. M. McDonald, D. M. D'Alessandro, R. Krishna, J. R. Long, *Chem. Sci.* **2011**, *2*, 2022.

(S6) P. Nugent, Y. Belmabkhout, S. D. Burd, A. J. Cairns, R. Luebke, K. Forrest, T. Pham, S. Ma, B. Space, L. Wojtas, M. Eddaoudi, M. J. Zaworotko, *Nature* **2013**, *495*, 80.

(S7) P.-Q. Liao, H. Chen, D.-D. Zhou, S.-Y. Liu, C.-T. He, Z. Rui, H. Ji, J.-P. Zhang and X.-M. Chen, *Energy Environ. Sci.*, 2015, **8**, 1011.

(S8) S. J. Datta, C. Khumnoon, Z. H. Lee, W. K. Moon, S. Docao, T. H. Nguyen, I. C. Hwang, D. Moon, P. Oleynikov, O. Terasaki and K. B. Yoon, *Science*, **2015**, *350*, 302-306.

(S9) T. M. McDonald, J. A. Mason, X. Kong, E. D. Bloch, D. Gygi, A. Dani, V. Crocella, F. Giordanino, S. O. Odoh, W. S. Drisdell, B. Vlaisavljevich, A. L. Dzubak, R. Poloni, S. K. Schnell, N. Planas, K. Lee, T. Pascal, L. F. Wan, D. Prendergast, J. B. Neaton, B. Smit, J. B. Kortright, L. Gagliardi, S. Bordiga, J. A. Reimer and J. R. Long, *Nature*, **2015**, *519*, 303-308.

(S10) N. T. T. Nguyen, H. Furukawa, F. Gándara, H. T. Nguyen, K. E. Cordova and O. M. Yaghi, *Angew. Chem. Int. Ed.*, **2014**, *53*, 10645-10648.

(S11) W. R. Lee, H. Jo, L.-M. Yang, H. Lee, D. W. Ryu, K. S. Lim, J. H. Song, D. Y. Min, S. S. Han, J. G. Seo, Y. K. Park, D. Moon and C. S. Hong, *Chem. Sci.*, **2015**, *6*, 3697-3705.
